# Specific Flavonoids and Their Biosynthetic Pathway in *Scutellaria baicalensis*

**DOI:** 10.3389/fpls.2022.866282

**Published:** 2022-03-03

**Authors:** Tianlin Pei, Mengxiao Yan, Yanbo Huang, Yukun Wei, Cathie Martin, Qing Zhao

**Affiliations:** ^1^Shanghai Key Laboratory of Plant Functional Genomics and Resources, Shanghai Chenshan Botanical Garden, Shanghai, China; ^2^National Key Laboratory of Plant Molecular Genetics, CAS Center for Excellence in Molecular Plant Sciences, Institute of Plant Physiology and Ecology, Chinese Academy of Sciences, Shanghai, China; ^3^John Innes Centre, Norwich, United Kingdom

**Keywords:** *Scutellaria baicalensis*, 4′-deoxyflavones, flavonoids, biosynthetic pathways, evolutionary mechanisms

## Abstract

*Scutellaria baicalensis*, is one of the most traditional medicinal plants in the Lamiaceae family, and has been widely used to treat liver and lung complaints and as a complementary cancer treatment in traditional Chinese medicine. The preparation from its roots, called “Huang Qin,” is rich in specialized flavones such as baicalein, wogonin, and their glycosides which lack a 4′-hydroxyl group on the B ring (4′-deoxyflavones), with anti-tumor, antioxidant, and antiviral activities. Baicalein has recently been reported to inhibit the replication of the COVID-19 virus. These 4′-deoxyflavones are found only in the order Lamiales and were discovered in the genus *Scutellaria*, suggesting that a new metabolic pathway synthesizing 4′-deoxyflavones evolved recently in this genus. In this review, we focus on the class of 4′-deoxyflavones in *S. baicalensis* and their pharmacological properties. We also describe the apparent evolutionary route taken by the genes encoding enzymes involved in the novel, root-specific, biosynthetic pathway for baicalein and wogonin, which provides insights into the evolution of specific flavone biosynthetic pathways in the mint family.

## Introduction

**FIGURE 1:**
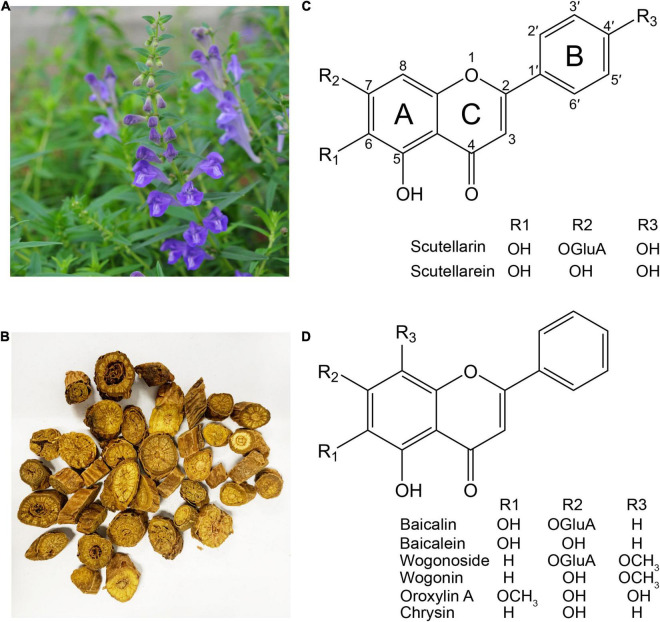
The medicinal plant *Scutellaria baicalensis* and its major flavones. **(A)** *S. baicalensis* plant. **(B)** The dried roots of *S. baicalensis* used in traditional Chinese medicine. **(C)** Structure of flavones produced from naringenin. **(D)** Structure of 4′-deoxyflavones derived from pinocembrin.

*Scutellaria baicalensis* Georgi (NCBI: txid65409) is a perennial herb belonging to the Lamiaceae family (Figure 1A). The dried root of *S. baicalensis* is known in China as “Huang (黄) Qin (芩)” (meaning golden herb) (Figure 1B), and was first recorded in *Shennong Bencaojing* (*The Classic of Herbal Medicine*) in around 200 AD. It is used for the treatment of bitter, cold, lung, and liver problems (Ma, 2013; Zhao et al., 2016a). It is indigenous to China, the Korean peninsula, Japan, Mongolia, and the Russian Federation, and has been cultivated worldwide as a medicinal plant (Shang et al., 2010). The potential medicinal activity of *S. baicalensis* was first recorded in *Bencao Gangmu* (*Compendium of Materia Medica*) published in 1593, with its root being used to alleviate the symptoms of diarrhea, dysentery, hypertension, hemorrhaging, insomnia, inflammation, and respiratory infections (Li, 1593). In recent decades, many studies have examined the potential hepato- and neuro-protective, antitumor, antimicrobial, anti-inflammatory, antioxidative, and antiviral effects of extracts of *S. baicalensis* (Wang et al., 2018).

Investigations into the pharmacological activities of *S. baicalensis* have focused mainly on the large amounts of specialized root-specific flavones (RSFs), such as baicalein, wogonin, and their glycosides baicalin and wogonoside. These RSFs lack a 4′-OH group on their B-rings (4′-deoxyRSFs) compared to the more typical 4′-hydroxyflavones such as scutellarein, which is widely distributed in aerial tissues of *S. baicalensis* (Figures 1C,D). The 4′-deoxyRSFs contribute most health benefits in *S. baicalensis* and specifically show a wide spectrum of antitumor activities (Table 1).

**TABLE 1:** The antitumor activities of 4′-deoxyRSFs from *Scutellaria baicalensis*.

Compounds	Potential clinical application	References
Baicalein	Prostate carcinoma, bladder cancer, head and neck squamous cell carcinoma, gastric cancer, colorectal cancer, oral cancer	Zhang et al., 2003; Bonham et al., 2005; Cheng et al., 2012; Wu et al., 2013; Wang et al., 2015; Mu et al., 2016; Chen et al., 2021
Baicalin	Mucoepidermoid carcinoma, prostate cancer	Chan et al., 2000; Xu et al., 2011
Wogonin	Gallbladder carcinoma, breast cancer, leukemia	Chung et al., 2008; Huang et al., 2010; Dong et al., 2011
Wogonoside	Acute myeloid leukemia	Chen et al., 2013

*Scutellaria baicalensis* is also an important ingredient of Qingfei Paidu Decoction (QPD). QPD is a new combined formula in traditional Chinese medicine (TCM) based on four classical formulae described in the *Shang Han Zabing Lun* (*Treatise on Cold Pathogenic and Miscellaneous Diseases*) (National Administration of Traditional Chinese Medicine, 2020). It is the only prescription in TCM recommended for treatment of mild, moderate, and severe cases of COVID-19 infection, and could also be used for critical patients according to their specific conditions (National Health Commission of the People’s Republic of China, 2021). Clinical data showed that COVID-19 related mortality was only 1.2% of the 2568 patients that received QPD compared with 4.8% of the mortality among 6371 patients that did not receive QPD (Zhang et al., 2021). This research also reported that the use of QPD could reduce 50% in-hospital, COVID-19-related mortality. Analysis of QPD shows it includes baicalin, the 4′-deoxyRSF from roots of *S. baicalensis* (Yang et al., 2020). Liu et al. (2021) reported that baicalein could suppress the replication of COVID-19 virus in Vero cells by inhibiting 3C-like protease, the main protease of severe acute respiratory syndrome coronavirus 2 (SARS-CoV-2). Other research gave similar results and confirmed the binding mode of baicalein with SARS-CoV-2 3C-like protease by X-ray protein crystallography (Su et al., 2020).

Flavonoids are large group of plant specialized metabolites and their biosynthetic pathways may evolve independently by convergent evolution which result in the production of these specialized bioactive flavones in widely diverged plant species (Wen et al., 2020). 4′-deoxyRSFs have a taxon-specific distribution and are also found sporadically in species outside the genus *Scutellaria* but in the order Lamiales, such as *Andrographis paniculate*, *Oroxylum indicum*, and *Plantago major* (Samuelsen, 2000; Chen et al., 2003; Li et al., 2007). They have also been reported in *Anodendron affine* and *Cephalocereus senilis* outside the order Lamiales (Qin et al., 1993; Tai et al., 2005). In recent years, taking advantage of newly developed genomic sequencing technologies, high-quality reference genome sequences for *S. baicalensis* have been published (Zhao et al., 2019; Xu et al., 2020). These were the first genome assemblies at chromosome-level resolution published for members of the family Lamiaceae. Based on the genomic information, notable progress has been made in elucidating the biochemical functions and evolutionary pathways of the genes encoding biosynthetic enzymes involved in the baicalein and wogonin metabolism in *S. baicalensis* (Zhao et al., 2016b; Zhao et al., 2018; Zhao et al., 2019; Xu et al., 2020).

## Flavonoid Metabolism in *Scutellaria baicalensis*

**FIGURE 2:**
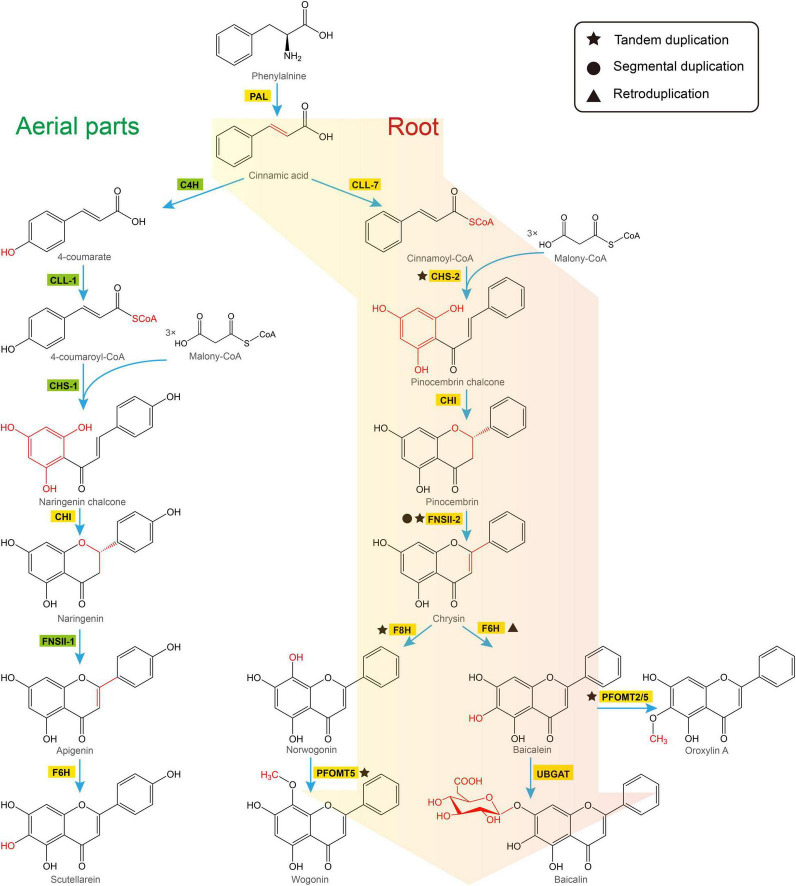
Two pathways responsible for biosynthesis of flavones in *Scutellaria baicalensis*. The classic flavone pathway makes scutellarein in aerial tissues (left hand), while 4′-deoxyRSFs are produced by a newly evolved pathway in roots (right hand). PAL, phenylalanine ammonialyase; C4H, cinnamate 4-hydroxylase; CLL-7, cinnamate-CoA ligase; CLL-1, 4-coumarate CoA ligase; CHS, chalcone synthase; CHI, chalcone isomerase; FNSII, flavone synthase II; F6H, flavone 6-hydroxylase; F8H, flavone 8-hydroxylase; PFOMT, flavonoid *O*-methyltransferases; UBGAT, baicalein 7-*O*-glucuronosyltransferase.

There are two distinct pathways in *S. baicalensis* responsible for the synthesis of flavones (Figure 2). In the aerial parts of the plant, naringenin is used as the precursor of scutellarein and scutellarin biosynthesis. Naringenin is synthesized from phenylalanine by general phenylpropanoid metabolism. Phenylalanine is converted to naringenin by phenylalanine ammonialyase (PAL), followed by ring hydroxylation by cinnamoyl 4 hydroxylase (C4H), activation by *p*-coumaroyl CoA ligase (4CL), condensation with 3 molecules of malonyl CoA by chalcone synthase (CHS), and isomerization by chalcone isomerase (CHI) (Lepiniec et al., 2006). Naringenin is then oxidized by flavone synthase II-1 (FNSII-1) to form apigenin, which is further hydroxylated and glycosylated to form scutellarein and scutellarin (Nagashima et al., 2000; Zhao et al., 2016b). Alternatively, the 4′-deoxyRSFs from roots of *S. baicalensis*, which include baicalein and wogonin, and their glycosides, are synthesized from a newly evolved pathway where cinnamic acid is activated by cinnamate-CoA ligase-like 7 (CLL-7) to form cinnamoyl CoA, which is then condensed with three molecules of malonyl CoA by a specific isoform of chalcone synthase (CHS-2), and isomerized by chalcone isomerase (CHI) to form pinocembrin, a flavanone without a 4′-OH group. Pinocembrin is then converted by a specialized isoform of flavone synthase II-2 (FNSII-2) to form chrysin, which serves as the precursor of other 4′-deoxyRSFs (Zhao et al., 2016b). Chrysin is decorated by flavone 6-hydroxylase (F6H) to produce baicalein, or by flavone 8-hydroxylase (F8H) and phenylpropanoid and flavonoid *O*-methyltransferases (PFOMT) to produce wogonin (Zhao et al., 2018; Zhao et al., 2019). These two RSFs are further glucuronidated by flavonoid 7-*O*-glucuronosyltransferases (UBGAT) to produce baicalin and wogonoside (Nagashima et al., 2000).

## Specific Function and Evolution of Enzymes Involved in the Biosynthesis of RSFs

### Phenylalanine Ammonialyase

Phenylalanine ammonialyase is one of the key enzymes acting between primary and secondary metabolism and catalyzes the deamination of L-phenylalanine to *trans*-cinnamic acid and ammonia (MacDonald and D’Cunha, 2007). Three full-length cDNAs encoding phenylalanine ammonialyase isoforms (*SbPAL1*, *SbPAL2*, and *SbPAL3*) were cloned from *S. baicalensis* using rapid amplification of cDNA ends (RACE) technology (Xu et al., 2010). Tissue-specific expression analysis indicated that the expression levels of *SbPAL1*, *SbPAL2*, and *SbPAL3* were highest in stems, leaves and roots, respectively. Besides, the transcripts of *SbPAL1*, *SbPAL2*, and *SbPAL3* were found to be up-regulated by MeJA and wounding treatments. To further confirm their function, *SbPAL1*, *SbPAL2*, and *SbPAL3* were overexpressed in *S. baicalensis* hairy roots by *Agrobacterium tumefaciens*-mediated transformation, and the transgenic hairy root lines produced higher baicalin, baicalein, and wogonin than the control hairy root line (Park et al., 2012). Comparative genome analysis revealed that the *PAL* gene numbers (five) in *S. baicalensis* were expanded compared to those (four) in *Scutellaria barbata*, another medicinal plant of *Scutellaria* genus accumulating RSFs. The specific expansion of these genes might have occurred via tandem duplication following the speciation of *S. baicalensis* and *S. barbata* (<13.28 Mya) (Xu et al., 2020).

### Cinnamate-CoA Ligase-Like

Cinnamate-CoA ligase-like are isoforms of 4CL which may activate cinnamic, benzoic, or fatty acid derivatives (Schneider et al., 2003; Shockey et al., 2003). Five full-length cDNAs of *CLL* genes were identified and cloned from *S. baicalensis* based on the root RNA-seq database (Zhao et al., 2016b). Phylogenetic analysis showed that SbCLL-1 and SbCLL-5 were grouped in the same clade as other known 4-CoA ligases and separated from the group of SbCLL-6, SbCLL-7, and SbCLL-8. Tissue-specific expression analysis suggested that *SbCLL-7* was expressed most highly in roots. The purified recombinant proteins of SbCLL-1 and SbCLL-5 could add CoA to cinnamic acid, 4-coumaric acid, and caffeic acid, while SbCLL-7 could add CoA only to cinnamic acid. Besides, SbCLL-7 had a substantially lower apparent *K*m value and higher *V*max/*K*m value for cinnamic acid compared to SbCLL-1 and SbCLL-5. Silencing of *SbCLL-7* in *S. baicalensis* hairy roots reduced the accumulation of baicalin, baicalein, and wogonoside compared to the empty vector controls. Comparative genome analysis revealed that *SbCLL-7* had syntenic homologs in *Salvia miltiorrhiza*, *Salvia splendens*, and *Sesamum indicum*, but these syntenic CLL-7s from other species of the Lamiaceae had no activity with cinnamic acid, 4-coumaric acid, or caffeic acid, indicating that the specific activity of SbCLL-7 for cinnamic acid is not shared by syntenic genes in species closely related to *S. baicalensis* (Zhao et al., 2019). Protein modeling of CLLs revealed that a more hydrophilic cysteine residue (C393) substituting a hydrophobic tryptophan (W) or leucine (L) residue might be responsible for SbCLL-7 binding cinnamate as a substrate. These point mutations in the *CLL* gene likely occurred after the divergence of *S. baicalensis*, from *S. miltiorrhiza* and *S. splendens* (<32.7 Mya) (Zhao et al., 2019).

### Chalcone Synthase

**FIGURE 3:**
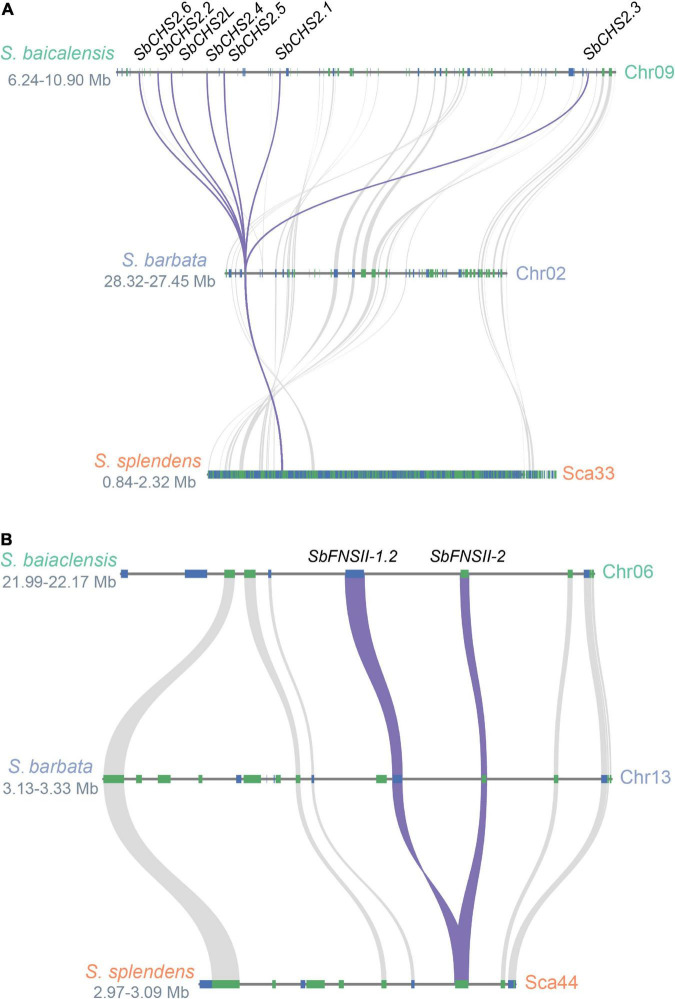
Evolutionary paths of *SbCHS2* and *SbFNSII-2*. **(A)** Syntenic analysis of *SbCHS2s* genes in *Scutellaria baicalensis*, *Scutellaria barbata*, and *Salvia splendens*. The multiplications of *SbCHS2* are mostly late at the species level for *S. baicalensis*. **(B)** Syntenic analysis of *SbFNSII-1* and *SbFNSII-2* genes in *S. baicalensis*, *S. barbata*, and *S. splendens*. The duplication of *SbFNSII-2* is earlier before the species divergence within *Scutellaria*.

Two full-length cDNAs of *CHS* genes (*SbCHS-1* and *SbCHS-2*) were identified and cloned from *S. baicalensis* based on the RNA-seq database (Zhao et al., 2016b). *SbCHS-1* is expressed specifically in flowers which may be responsible for the biosynthesis of classic flavones and anthocyanins. *SbCHS-2* is highly expressed in roots and encodes an enzyme involved in the formation of pinocembrin chalcone and responsible for RSF biosynthesis (Zhao et al., 2016b). Based on genome analysis, a tandem multiplication event occurred in the genomic region of *SbCHS-2*, which produced five adjacent gene copies (*SbCHS2.1-SbCHS2.6*). This example of gene amplification might increase transcript and protein dosage and drive stronger flux along the RSF biosynthetic pathway (Zhao et al., 2019). The neofunctionalization of SbCHS-1 and SbCHS-2 followed the divergence of the *Salvia* and *Scutellaria* lineages (∼32.7 Mya). The ancestral *CHS* gene was duplicated in *S. baicalensis*, with one copy (*SbCHS-1*) moving to pseudochromosome 3, while the other copy (*SbCHS-2L*) remained on pseudochromosome 9 (∼19 Mya). *SbCHS-2L* was further duplicated (*SbCHS2.3*) around 12 Mya, and was then amplified by tandem duplications to produce the other *SbCHS-2* genes accompanied by neofunctionalization probably within the last 1 Mya (Figure 3A). In *S. barbata*, *CHS-2* has not been amplified to the same extent as in *S. baicalensis* (Xu et al., 2020). This confirms that most of the amplifications of *CHS-2* occurred very recently (Zhao et al., 2019), perhaps linked to domestication of *S. baicalensis* through its use in TCM.

### Chalcone Isomerase

One full-length cDNA encoding SbCHI was cloned from *S. baicalensis* using rapid amplification of cDNA ends (RACE) technology (Park et al., 2011). Tissue-specific expression analysis indicated that the expression levels of *SbCHI* were highest in roots, whereas lower expression levels were detected in the flowers, stems, and leaves. The transcripts of *SbCHI* were found to be up-regulated by MeJA and wounding in suspension cell cultures of *S. baicalensis*. Overexpression of *SbCHI* in *S. baicalensis* hairy roots enhanced the production of baicalein, baicalin, and wogonin, while *SbCHI*-silenced hairy root lines showed reduced accumulation of baicalein and baicalin (Park et al., 2011). There is only one locus encoding SbCHI and its activity is shared by both aerial and RSF biosynthetic pathways (Zhao et al., 2016b; Zhao et al., 2019).

### Flavone Synthase

Two different types of FNS enzyme (FNSI and FNSII) are responsible for the conversion of flavanones to flavones by introducing the double bond between the C2 and C3 positions in *S. baicalensis*. FNSI belongs to the 2-oxoglutarate-dependent dioxygenase (2OGD) superfamily and is found only in species of the Apiaceae (Umbelliferae) (Kawai et al., 2014), while FNSII is a cytochrome P450 (CYP450) monooxygenase widely distributed in angiosperms (Martens and Mithöfer, 2005). Generally, FNSI and FNSII catalyze the conversion of flavones with a 4′-OH group, such as naringenin, eriodictyol, and liquiritigen into flavones (Britsch, 1990; Fliegmann et al., 2010). Zhao et al. (2016b) reported a neo-functional FNSII from *S. baicalensis* (SbFNSII-2) which is required for the biosynthesis of specialized 4′-deoxyRSFs. *SbFNSII-2* is highly expressed in roots and is induced by MeJA. It can convert only pinocembrin to chrysin in both *in vivo* and *in vitro* assays. RNAi silencing of *SbFNSII-2* in hairy roots of *S. baicalensis* significantly reduced the accumulation of baicalin, wogonoside, and baicalein. *S. baicalensis* also produces an isoform of FNSII (SbFNSII-1) that converts naringenin to apigenin in aerial parts of the plant. In *S. baicalensis* there are two *SbFNSII-1* loci which arose as part of a segmental duplication which occurred after the divergence of the Lamiaceae (<42.7 Mya). However, just one *SbFNSII-2* locus is present in the *S. baicalensis* genome suggesting that *SbFNSII-2* arose by tandem duplication of *SbFNSII-1*, and neofunctionalization following the divergence of genus *Salvia* and the genus *Scutellaria* (<32.7 Mya) (Zhao et al., 2019). In *S. barbata*, one gene orthologous to *FNSII-2* can be detected in a tandem duplication of *FNSII-1* and *FNSII-2* in the region collinear between *S. baicalensis* and *S. barbata* (Figure 3B) (Xu et al., 2020). This would place the *FNS-II* duplication earlier than the species divergence within the genus *Scutellaria* and indicate the conserved evolution of 4′-deoxyflavones in the genus *Scutellaria*.

### Flavone Hydroxylase

Two CYP450 family members in *S. baicalensis* are responsible for the hydroxylation of the 6- and 8-positions of the flavones (SbF6H and SbF8H, respectively). These two enzymes convert chrysin to baicalein and norwogonin, respectively (Zhao et al., 2018). SbF6H (CYP82D1.1) can use both 4′-hydroxyflavones and 4′-deoxyflavones (such as apigenin and chrysin, respectively) to produce scutellarein and baicalein in aerial parts and roots, respectively. RNAi silencing of *SbF6H* resulted in a significant reduction in the levels of baicalin and baicalein levels but promoted the accumulation of chrysin glycosides in hairy roots. SbF8H (CYP82D2) accepts only chrysin as its substrate to produce norwogonin, and exhibits only very minor 6-hydroxylation activity. Silencing of *SbF8H* reduced the levels of wogonin and wogonoside, but slightly increased baicalin levels. Structural modeling revealed that several amino acid substitutions contribute to the substrate binding in different orientations in the active site of SbF6H and SbF8H, resulting in their divergent catalytic activity (Zhao et al., 2018). In *S. baicalensis* there is only one *SbF6H* locus on pseudochromosome 5 which was likely derived from multiplication of CYP82D genes on pseudochromosome 1 and this gene acquired its new position by retrotransposition, while *SbF8H* underwent tandem multiplication and neofunctionalization (Zhao et al., 2019).

### *O*-Methyltransferases

*O*-methyltransferases are responsible for the transformation of a methyl group from S-adenosyl-L-methionine (SAM) to a hydroxyl group on their substrate. *O*-methylation contributes to the structural diversity of flavonoids and modifies their solubility, stability, and bioactivity (Ibrahim and Anzellotti, 2003). In *S. baicalensis*, two types of OMT are involved in the biosynthesis of root-specific 4′-deoxyflavones. Type II OMTs are Mg^2+^-dependent and also known as phenylpropanoid and flavonoid OMTs (PFOMTs), which are reported to transfer methyl groups to flavones with adjacent hydroxyl groups on their aromatic rings. In *S. baicalensis* roots, SbPFOMT2 and 5 can efficiently *O*-methylate the C6, C8, and C3′ positions of flavones to form mono-methoxyflavones oroxylin A, wogonin, tenaxin II, and chrysoeriol, respectively (Zhao et al., 2019; Cui et al., 2021). Three type I OMTs from *S. baicalensis*, referred to as flavonoid OMTs (SbFOMTs), have been found to decorate hydroxyl 4′-deoxyflavones. SbFOMT3 is a 7-OMT that converts baicalein to 7-methoxybaicalein, while SbFOMT6 is a 7-OMT that can use both baicalein and norwogonin as substrates. SbFOMT5 can methylate hydroxyl groups of baicalein on the C5, C6, and C7 positions. Combination of SbPFOMT5 and SbFOMT6 or SbPFOMT5 plus SbFOMT5 can produce skullcapflavone I and tenaxin I, respectively, in yeast. These root methoxylated flavones exhibit stronger activities than baicalein in inducing apoptosis of human cancer cell lines (Cui et al., 2021). In addition, *SbPFOMT5* has undergone recent tandem duplication and neofunctionalization (Zhao et al., 2019).

### Baicalein 7-*O*-Glucuronosyltransferase

Glycosylation is an important decoration for flavones in the stabilization and enhancement of their solubility in water (Bowles et al., 2005). Baicalin is a 7-*O* glucuronide derived from baicalein, catalyzing by a UBGAT in *S. baicalensis* (Nagashima et al., 2000). This enzyme is also responsible for glucuronidation of the 7-OH group of flavones with *ortho*-substituents, such as wogonin and scutellarein (Nagashima et al., 2000).

## Conclusion and Future Directions

In summary, the 4′-deoxyRSF biosynthetic pathway emerged after divergence of the genera *Scutellaria* and *Salvia* (< 32.7 Mya), and likely evolved by point mutation (*SbCLL-7*), tandem multiplication and neofunctionalization (*SbCHS-2*, *SbFNSII-2*, *SbF8H*, and *SbPFOMT*), segmental duplication (*SbFNSII-2*), and retroduplication (*SbF6H*). Specifically, *SbCHS-2* underwent several rounds of amplification: the earlier duplications (*SbCHS2L* and *SbCHS2.3*) occurred before the species divergence within *Scutellaria*, while tandem multiplications (*CHS-2.1*, *CHS-2.2*, *CHS-2.4*, *CHS-2.5*, and *CHS-2.6*) occurred later at the species level, in *S. baicalensis*.

Specialized metabolites from plants have served in history as the main source of medicines for humans when challenged by pandemics (Weng, 2020), such as the COVID-19 virus, which has infected 280 million people and has killed over 5.4 million people at the time of writing this manuscript (World Health Organization^1^). Baicalein from the roots of *S. baicalensis* was reported to be an effective inhibitor of the COVID-19 virus (Su et al., 2020; Liu et al., 2021). So far, the complete biosynthetic pathway of baicalein has been elucidated, and the production of baicalein directly from glucose by *Escherichia coli*-fed batch fermentation can reach 214.1 mg/L (Ji et al., 2021). These studies provide a model for discovering and utilizing medicinal plants for development of therapies. Nevertheless, enzymes involved in the modification of the B-ring of some flavones found in *S. baicalensis* roots, such as tenaxin I, skullcapflavone I, rehderianin I, and viscidulin III, have not yet been identified. These flavones are hydroxylated on the 2′, 3′, 5′, and 6′ positions of their B-ring and some of them (tenaxin I and skullcapflavone I) exhibit stronger cytotoxicity than baicalein in cancer cells apoptosis assays (Cui et al., 2021), which has inspired us to explore further the biosynthetic pathways as well as their clinical applications.

*Scutellaria* includes about 350 species around the world, and plants of this genus have been widely used in traditional medicine for thousands of years. For example, *S. barbata*, a plant native to southern China, has been used to cure pain and swelling of throat, edema and hemorrhoids as well as its prescription for cancer treatment; *Scutellaria galericulata* and *Scutellaria lateriflora* are used as sedative/nerve tonics in Europe and North America; and *Scutellaria indica* has been employed for detoxification and promoting blood circulation effects in China, South Korea, and India. In addition to flavones, *Scutellaria* plants are rich in diterpenoids or diterpenoid alkaloids. These compounds exhibit excellent antitumor activities and their biosynthetic pathways are not fully understood (Shang et al., 2010). Rapidly developed genome sequencing, metabolite profiling, gene editing, and machine learning (AlphaFold) technologies will help us study the biosynthesis of active ingredients in *Scutellaria* plants as well as their diversification and evolution.

## Author Contributions

TP collected literatures and wrote the manuscript. MY performed the evolutionary analysis of *SbCHS2* and *SbFNSII-2*. YH and YW provided the image of *S. baicalensis* plants. QZ and CM revised the manuscript. All authors read and approved the final manuscript.

## Conflict of Interest

The authors declare that the research was conducted in the absence of any commercial or financial relationships that could be construed as a potential conflict of interest.

## Publisher’s Note

All claims expressed in this article are solely those of the authors and do not necessarily represent those of their affiliated organizations, or those of the publisher, the editors and the reviewers. Any product that may be evaluated in this article, or claim that may be made by its manufacturer, is not guaranteed or endorsed by the publisher.

## Footnotes

1
covid19.who.int/
